# Implementing a larviciding efficacy or effectiveness control intervention against malaria vectors: key parameters for success

**DOI:** 10.1186/s13071-018-2627-9

**Published:** 2018-01-24

**Authors:** Christophe Antonio-Nkondjio, Nino Ndjondo Sandjo, Parfait Awono-Ambene, Charles S. Wondji

**Affiliations:** 1 Laboratoire de Recherche sur le Paludisme, Organisation de Coordination pour la lutte Contre les Endémies en Afrique Centrale (OCEAC), P.O. Box 288, Yaoundé, Cameroon; 2 Vector Group Liverpool School of Tropical medicine Pembroke Place, Liverpool, L3 5QA UK; 3 Montreal University School of Public Health, 7101 Av du Parc, Montréal, QC, H3N Canada; 4 SPatial HEalth REsearch Lab (SPHERE LAB), Montreal University Hospital Research Center (CRCHUM), 900 Rue Saint-Denis, Montréal, QC, H2X 0A9 Canada

**Keywords:** Larviciding, Clusters randomized trials, Malaria, Vector control, Study design, Outcomes, Implementation

## Abstract

During the last decade, scale-up of vector control tools such as long-lasting insecticidal nets (LLINs) and indoor residual spraying (IRS) contributed to the reduction of malaria morbidity and mortality across the continent. Because these first line interventions are now affected by many challenges such as insecticide resistance, change in vector feeding and biting behaviour, outdoor malaria transmission and adaptation of mosquito to polluted environments, the World Health Organization recommends the use of integrated control approaches to improve, control and elimination of malaria. Larviciding is one of these approaches which, if well implemented, could help control malaria in areas where this intervention is suitable. Unfortunately, important knowledge gaps remain in its successful application. The present review summarises key parameters that should be considered when implementing larviciding efficacy or effectiveness trials.

## Background

The large scale implementation of vector control measures across Africa over the last decade has permitted a decrease in malaria transmission and malaria burden. According to recent reports by the World Health Organization, up to 57 countries across the world have reduced their malaria cases by 75% [1]. Reported malaria mortality rates have fallen by 66% among all age groups and by 71% among children under five [1, 2]. Some countries across the world have over the last decade reported elimination of the disease and many more are advanced in this goal [1, 3]. Yet the disease remains largely prevalent particularly in sub-Saharan Africa with over 3.2 billion people still at risk of malaria. In 2015 over 214 million new malaria cases were reported with 438,000 deaths [1]. For countries still facing strong challenges affecting malaria control such as, rapid expansion of insecticide resistance, outdoor malaria transmission, change in vector feeding and biting behaviour, urban malaria transmission or transmission in hotspots areas, the use of additional control measures such as larval control in an integrated control approach could appear decisive for the control and elimination of the disease. Several compounds including synthetic organic chemicals, bacterial larvicides, spinosyns, insect growth regulators can be used as larvicides [4]. Unfortunately, little information is available on the efficacy and effectiveness of the majority of these larvicides in African settings [5], preventing the use of these tools by control programmes [5]. The limited use of larval control tools for malaria vector control could also be attributed to the poor knowledge on methods of implementing and monitoring the intervention, the assumed high operational costs of this intervention, the intensive labour required for its implementation and the short residual effect of previous larvicides formulations [4]. Because larval control targets mosquito at the larva stage it kills both outdoor and indoor biting mosquitoes and could be a good supplement to existing indoor base interventions such as LLINs or IRS used as first line interventions. Larviciding with the use of microbials such as *Bacillus thuringiensis* or *Bacillus sphaericus* or a combination of the two have shown to be particularly efficient for controlling malaria vectors in different epidemiological settings [6–10]. New formulations in granules, microcapsules or briquettes presenting long residual effects or efficient against several mosquitoes are now available and this, by reducing the operational cost of this intervention, could encourage more programmes to step in and use larval control frequently as an additional measure for controlling malaria transmission. This would be particularly the case in urban settings where intervention could be cost-effective [10, 11]. Yet according to recent reports, the number of unbiased studies on the efficacy or the effectiveness of larval control trials across Africa is still insignificant and makes it difficult to draw generalized conclusions on this intervention efficacy for malaria vector control [5, 12, 13]. The limited number of unbiased studies could come from the imperfect knowledge on the procedures to design, implement and assess larval control efficacy or effectiveness trials. With the growing requirement for clusters randomised trials (CRT) in vector control efficacy or effectiveness trials, it becomes essential to recall important guidelines to insure that, strong evidence is reported from these studies. The aim of this paper is to present a simple review of key parameters to consider when implementing malaria vector larviciding efficacy or effectiveness control interventions. The present review will focus exclusively on larviciding implementation which is defined by the World Health Organization [4], as the regular application of biological or chemical insecticides in mosquitoes breeding habitats.

## Larvicides for malaria vector control

According to the World Health Organization [4], several compounds including oils and surface films, synthetic organic chemicals, bacterial larvicides, spinosyns and insect growth regulators can be used for larviciding.

### Synthetic organic chemicals

This group includes all insecticides (organochlorines, pyrethroids, organophosphates) that can be used for larval control. Insecticides are neurotoxic compounds which kill insects by interfering with the normal transmission of nerve impulses [14] (Table 1). DDT has been largely used across the world for indoor residual spraying and larval control [15] but this compound is no longer used in most parts of the world because of its persistence in the environment and in organisms tissues, and its harmful effect on humans and non-targets [16, 17]. Pyrethroids were also used for larval control [4, 18, 19] but due to risks of selection of insecticide resistance at larval stage which could affect the performance of treated bednets, they are no longer recommended [4]. Organophophates (temephos, fenthion) are the only class of insecticides approved for larval control because of their high efficacy and low persistence in the environment [4]. They have been used successfully for mosquito and black fly control [20–22]. New insecticide candidates deriving from plants extracts or essential oils are now under study [23–25]. Many of these have been reported to cause high larval mortality after ingestion or growth inhibiting effects [25]. Yet just a few have undergone chemical characterization and none have so far undergone field evaluations [23, 25].

**Table 1:** Summary of characteristics of compounds used as larvicides

Larvicides	Mode of action	Advantages	Limitations	Residual effect
Bacterial larvicides	Induce the formation in larval midgut of a toxic pore that kill the mosquito by interrupting feeding and homeostasis	Harmless to most aquatic non-target organisms and humans, effective against insecticide resistant mosquitoes	Previous formulations had limited residual effect, require larvae to feed on, not active on late instar larvae and pupae	2 weeks previous formulations/up to 6 months for new formulations
Spinosyns	Toxic after ingestion and neurotoxic effect (bind to GABA and the nicotinic acetylcholine receptors and stop the normal transmission of nerve impulse and induce insect death).	Efficient against a large spectrum of species safe to non-target organisms, effective against insecticide resistant mosquitoes	Also used in agricultural, limited residual effect requiring frequent re-treatments	Short (1 to 3 weeks)
Petroleum products	Direct toxicity after ingestion or by contact reducing the mobility and prevent larvae from breathing causing suffocation and larval death	Control all mosquitoes, cheaper, easy to acquire, mosquito cannot develop resistance to the compound	Toxic to non-target species, frequent retreatment required, can be dispersed by wind, rain, vegetation or animals	Short (1 to 3 week)
Monomolecular surface films (MMF)	Prevent larvae from breathing and induce suffocation and larval death	Biodegradable, spread spontaneously over large water surface, safe to non-target organisms, mosquito cannot develop resistance to the compound	Can be dispersed by wind, rain, vegetation or animals	Short (up to 1 week)
Insect growth regulators	Prevent the development of larvae to adults or kill larvae when moulting	Efficient against several mosquito species, long residual effect, effective at low dosage, effective against insecticide resistant mosquitoes	Difficult to monitor if sites have been treated or not, toxic for non-target aquatic organisms	Long (3 to 6 months)
Essential oils and plant extracts	Toxic after ingestion or growth inhibiting effects	New compounds, could improve control of resistant mosquitoes	Not well characterised, no efficacy trial conducted, difficult to produce large quantities	Short
Synthetic chemicals	Neurotoxic compounds which kill insects by blocking the normal transmission of nerve impulses	Easy to implement, effective in polluted habitats	Can select for insecticide resistance, harmful to non-target organisms, frequent re-treatment required, only organophosphates approved for larval control	Long (several weeks)

### Oils and surface films

Oils and surface films deriving from petroleum oil or isotearyl alcohol could be used for larval control. The application of petroleum oils in water is considered as one of the ancient modes of control of mosquito larvae [26]. From the 1920s through to the 1960s, petroleum oils have been frequently used for mosquito larvae control in the Americas and in India [26–29]. Petroleum products such as kerosene, petrol and engine oils are still used in local communities in West Africa for controlling the mosquito burden [30, 31]. Petroleum products are known to be active against mosquito larvae through two mechanisms: direct toxicity and suffocation [32]. However these oils present a certain number of limits such as the non-uniform spreading of oils requiring soluble surface active ingredients for their spreading or their dispersion by wind or rain [33–35], their toxicity to non-target organisms and to the environment [36, 37]. Derivatives such as monomolecular surface films (MMF) have been developed over recent years to replace petroleum oils and serve as larvicides and pupicides for mosquitoes [32, 38]. These products are biodegradable and spread spontaneously over large water surfaces to form an ultrathin film preventing larvae from breathing and therefore, induce suffocation and the death of larvae [32, 38]. Their mode of action against mosquito larvae is physical rather than chemical. They can affect both the larval and the adult stages of the mosquito [39]. MMF have been shown to be relatively safe to non-target and aquatic organisms including humans. Several formulations are available and await large scale field evaluation [32, 38]. Yet the use of MMF in larval control is subject to environmental challenges such as rainfall, wind and vegetation which could induce dispersion and patchy distribution of MMF layers on water surface [40].

### Spinosyns

Spinosyns are products from the fermentation by *Saccharopolyspora spinosa* comprising spinosyn A (as the main component) and spinosyn D (as the minor component). These compounds act on the central nervous system by binding to the nicotinic acetylcholine receptor and GABA (gamma-aminobutyric acid) receptors interrupting the normal transmission of nerve impulse [41]. Spinosyns act through direct contact or after oral ingestion and are harmless to fish, mammals and birds [41]. Due to the large spectrum of species that could be targeted by these compounds including Lepidoptera, Diptera, cockroaches, spider mites, leafhoppers and various insect orders [41–45], several applications of spinosyns in veterinary, agriculture or human health have been reported [41, 45, 46]. Spinosyns are still not widely used for vector control [47], but are used for pest control in agriculture [41]. Their use for pest species control started in 1997. Few cases of resistance have so far been reported in some pest species and *Drosophila melanogaster* [48, 49].

### Bacterial larvicides

These include organisms producing insecticidal crystal proteins toxic for the mosquito which is particularly the case for strains of *Bacillus thurigiensis israelensis* (Bti), *Bacillus sphaericus*, *Brevibacillus laterosporus* and *Clostridium bifermentans* [50, 51]. However only *Bacillus thurigiensis israelensis* and *Bacillus sphaericus* are commonly used for vector control because of their high toxicity to mosquitoes and broad spectrum of target species [4]. Bacterial larvicides act after ingestion by binding to specific receptors in the larval midgut and induce spore formation and larval death [50, 51] (Table 1). Due to their specific mode of action, bacterial larvicides are harmless to most aquatic non-target organisms and humans and have been frequently used for larval control in Africa [6, 9, 52] and across the world [7, 44, 53]. Previous formulations requested frequent retreatments whereas new formulations have a longer residual effect reaching up to six months and are effective in organically polluted sites and on a large spectrum of mosquito species [4, 10, 11].

### Insect growth regulators (IGR)

Insect growth regulators include anti-juvenile hormone agents which prevent the development of larvae or pupae into adults (e.g. methoprene and pyriproxyfen) and chitin synthesis inhibitors which kill larvae during moulting (diblubenzuron and triflumeron) [4]. Some IGR such as pyriproxyfen were also shown to have ovicidal activity inhibiting egg hatching and development [54, 55] or reducing the reproduction potential of adult mosquitoes [56]. Most of these compounds have a longer residual effect lasting up to six months (Table 1). They affect a large number of species. Most laboratory tests conducted so far indicated high efficacy of the majority of compounds on *Aedes*, *Culex* or *Anopheles* species [55–57]. However, their potential for malaria vector larval control is still under investigation in Africa [38, 58–60]. A major limit to the use of IGR is the difficulty to monitor the effectiveness of field treatments [4].

## Important guidelines for the implementation of larval control interventions

The World Health Organization [12] interim position on the use of larval source management in sub-Saharan Africa states that anti-larval measures can be cost-effective in settings where breeding sites are few, fixed and findable. Larviciding in sub-Saharan Africa should be considered only as a supplement to the core interventions (ITNs or IRS). As condition for its success, larviciding need to be implemented in sites where malaria transmission is low to moderate, where there is a high coverage with first line interventions such as IRS or LLINs. Larviciding is most likely to be appropriate for urban settings because the conditions stated above are more likely to be met and because the high population density make the intervention cost effective [4]. Larviciding is an intervention that needs to conform to local environmental conditions. In certain circumstances when first line control measures are not performing well in cases of either high prevalence of insecticide resistance, high transmission by outdoor biting mosquitoes, a change in vector feeding and biting behaviour or for controlling mosquito burden, the use of larviciding could prove to be appropriate [12].

Conducting a vector control intervention will require the researcher to decide whether he wants to implement an efficacy or an effectiveness control trial. According to Flay et al. [61], an efficacy trial refers to the beneficial effects of a programme or a policy delivered under optimal conditions of delivery, whereas effectiveness trials refer to the effects of a programme or policy under more real-world conditions. This means that in an efficacy trial, which could be considered as a pilot study, the researcher will have as main objective to assess the performance of the intervention when it is not significantly affected by bias or confounding factors (under ideal conditions of delivery). On the other hand, in an effectiveness study, the researcher will assess whether the intervention can be sustainable beyond the pilot stage or can be extended to a larger geographical scale, he/she can also focus on factors insuring performance, sustainability and success of the intervention. In either case, implementing a successful larviciding or vector control trial will require that:

- A good study design is developed to address the research question.
- A good sample size is defined for the study.
- A high quality of programme implementation and monitoring is undertaken.
- Unbiased methods and standardized procedures are used for data collection, processing and analysis.
- Appropriate statistical approaches are used for data analysis.
- Consistent positive effects without iatrogenic effect are recorded.
- Long-term follow up are conducted before releasing concluding remarks.

This requires that everything is well thought in advance when designing the study to ensure robustness of concluding remarks.

## Study design: use of Cluster Randomized Trial (CRT) design

Although individual or collective measures can be used as preventive measures for vector-borne diseases, evaluation of these interventions are usually conducted at the community level meaning that cluster randomized trials are more appropriate for this purpose; these are considered as the most appropriate method to evaluate preventive interventions because they generate statistically unbiased estimates and reduce the risk of selection bias [62]. A two-arm parallel CRT design is actually the most popularly used method in clinical trials and in vector control interventions [6, 62]. In this type of study design, clusters are individually allocated as treated or untreated using either a random table or a computer assisted programme. In case cluster allocation is not random, one needs to adjust for pre-intervention differences to minimise potential bias. This was done in the larval control trial in Dar es Salaam Tanzania where the authors chose to allocate clusters as treated progressively according to the ward supervisors and ward-based corps’ ability to collect, understand, use and submit high quality data during baseline studies [8, 9].

Because a loss of efficiency could occur when a two-arm parallel CRT design is used, or due to the imbalance of baseline outcomes distribution requiring statistical adjustments, many investigators prefer matching clusters into pairs based on the similarity of baseline characteristics before one in each pair is randomly assigned as treated or untreated [63, 64]. This method is considered to greatly reduce imbalances and increase the power of detecting causal effect estimations [63–65]. Some larval control trials have been conducted using this design [10, 63]. Complex designs can also be adopted depending on the characteristics of factors to assess [66].

Randomized units arbitrarily defined as geographical areas or clusters could be either define around key parameters of the study such as hospitals, rivers, hot spots or just refer to a community, a district or a village as was done in larval control trials conducted in Kenya, Tanzania and Gambia [6, 8, 9, 52]. The boundaries of each unit or cluster need to be clearly defined to avoid overlapping and bias. When designing clusters, it is always important as for clinical trials, to define inclusion and exclusion criteria. Making a list of what could be considered as inclusion or exclusion criteria could be helpful for cluster design and selection.

## Research questions

Conducting a larval control trial requires as a prerequisite the definition of a clear research question that will enable good assessment of the intervention impact. Additionally, drafting clear hypotheses and the target (awaited impact) helps to determine what outcome should be selected and measured. In the larval control trial in western Kenya, Fillinger et al. [6] assumed their intervention will reduce by 33% the incidence of new *Plasmodium* infections in children. Clearly defining the target enables identification of variables that could be used as primary outcome.

## Primary and secondary outcomes

The success of an intervention depends on the choice of a set of reliable and sensitive outcomes capable of detecting the impact of the intervention. In a study, depending on the objectives, there is always a primary outcome and several secondary outcomes. A primary outcome represents the main variable enabling appreciation of the overall impact of the intervention it is also used for estimating the sample size. In vector control trials investigators are tempted to use exclusively entomological outcomes [62], because these enable assessment of the intervention impact when there are several interventions implemented in the same site [4]. In a systematic review assessing community effectiveness of temephos for dengue vector control, out of 27 studies analysed none assessed epidemiological outcomes [67]. Using both entomological and epidemiological outcomes could provide a better understanding of the epidemiological impact of the trial. Epidemiological outcomes are considered as the best predictors to use for preventive interventions because they allow a good assessment of the intervention efficacy in protecting the human population [62]. Epidemiological variables directly collected from the field such as the prevalence or the incidence of new malaria cases are considered as good indicators and were used in several trials [6, 52]. Entomological variables, directly related to disease transmission can provide a better understanding of the epidemiological impact of the intervention [62]. Yet some outcomes such as the Entomological Inoculation Rate, although providing detailed information on the transmission of the disease from mosquitoes to humans, it is not recommended to be used as a primary outcome because it is an indirect measure of field collected variables in this case it will be advisable to use adult mosquito density as done in previous trials [6, 52].

Secondary outcomes can be as many as possible and they must be reliable and sensitive. They can be either direct or indirect measures from the field depending on the study objectives, and different sets of variables could be measured. In larviciding studies conducted in Kenya, Tanzania and in the Gambia, the authors collected variables from households, entomological and clinical surveys [6, 9, 52].

## Sample size calculations

Calculating the sample size comes from the need to have sufficient statistical power to detect differences between the intervention and the control groups (minimising the risk of type II error, the failure to detect a significant impact of the intervention when there is truly one). Using a smaller sample size will have a low precision as a direct consequence, whereas larger sample sizes will result in the increased precision of measured parameters 95% confidence interval. The lack of an effect during an intervention could come from the fact that the study is underpowered. This has been the case for many larviciding studies conducted so far in Africa using very few clusters [5]. Studies using a minimum 80% power for sample size estimation are generally considered reliable for entomological studies or clinical trials [68]. Also important is defining the significance level which is the threshold for statistically significant outcomes. The commonly used value in research is α = 0.05. If a large number of comparisons are undertaken, the Bonferroni correction should be applied to determine the significance threshold to avoid Type 1 error (false significant results) [69].

**Fig. 1:**
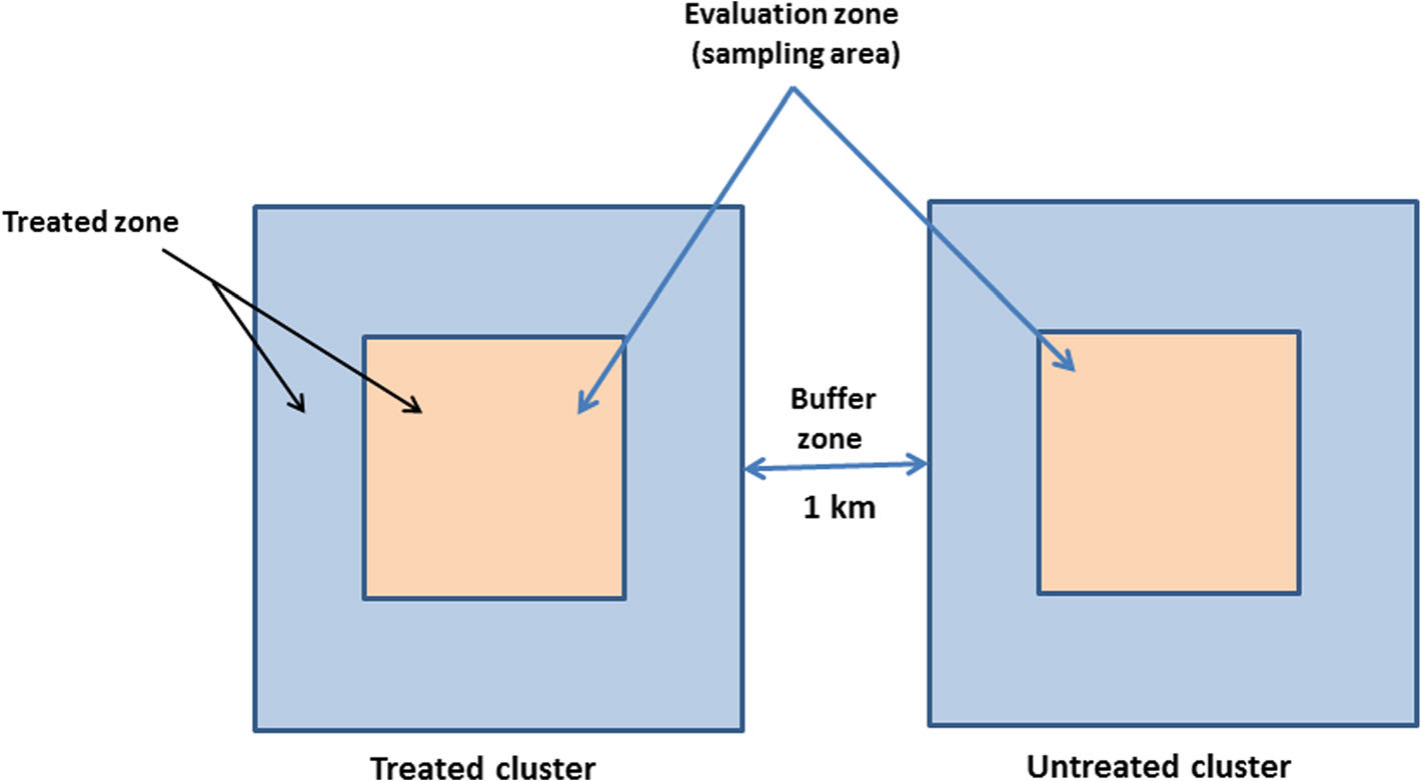
Description of a cluster design during a larval efficacy trial in order to minimise contamination due to mosquito spillover

After defining the number of clusters, it is important to determine the size of each cluster in order to minimise as much as possible contamination or spillover effects. One strategy is, for example, to design clusters very large and to sample and evaluate the intervention at the centre of the cluster (Fig. 1). This design was adopted in the Gambia, where Majambere et al. [52] used large size clusters and sampled and evaluated their intervention only in villages situated at the centre of each cluster. However, the use of very large clusters could require an increased sampling effort and could affect implementation and monitoring of the intervention particularly if inspections and treatment are done manually and if breeding sites are numerous. According to Hayes & Moulton [70] the use of a high number of smaller clusters is better than using few big ones. A minimum distance between clusters needs to be defined in order to minimise as much as possible contamination due to mosquito spillover from untreated to treated sites. Most larviciding trials considered a minimum distance of 1 km between adjacent clusters as enough to minimise contamination [6, 63]. In Kenya a reduction in the risk of acquiring malaria infection of up to 56% was recorded between treated and untreated clusters situated 1 km apart [6]. Moreover, because the measurement of epidemiological outcomes could be subject to contamination due to population movements from untreated to treated sites, it is recommended to use a less mobile population such as children as done in previous trials [6, 52].

Additionally, calculating the sample size will require having prior information on the prevailing situation before the intervention to set a target (reduction level to be achieved). This information could be retrieved from previous reports conducted in the area [6]. In case the information is not available, a preliminary study can be conducted to collect this information. For CRT, sample size calculations can be undertaken using formulas provided in Haye & Bennett [68] or elsewhere [66]. Yet after sample size calculations, a certain number of simulations need to be done to test the robustness of sample size estimations [68]. Simulations can be undertaken to verify whether the sample size is appropriate in case of variation of the intercluster correlation coefficient k, in case of violation of baseline assumptions, in case of variation of clusters sizes or, in case the study achieves a target lesser than estimated. It is always recommended to add 1 or 2 extra clusters per treatment group to take into consideration lost to follow up or unforeseen changes, which might be responsible for imbalance between groups and systematic bias [66, 69].

### Intracluster correlation coefficient (ICC)

The ICC could be defined as the measure of the homogeneity of observations within clusters of a random effect with respect to the dispersion of these observations between clusters. It compares the variance within clusters with the variance between clusters. It is calculated by dividing the variance between clusters by the sum of variance within and between clusters [71]. Estimates of the ICC are very useful for sample size calculations.

### Intercluster correlation coefficient

This variable (k) measures the level of variation between clusters and is very important for sample size calculation. Because the variable is not readily available when conducting a trial, Hayes & Bennett [68] recommend to examine the required sample size for various plausible values of k. Data collected from different field trials suggest that k is often less or equal to 0.25 or does not normally exceed 0.5 for most health outcomes [68].

## Data analysis and clustering adjustments

Clustering correlated data arise when there is a group structure to the data or when the data present a hierarchy with multilevel units. For example, in a longitudinal larval control study, the repeated measurements obtained from a single breeding site at different months or seasons represent level 1 and the breeding sites represent level 2. Clustering adjustment enables correction, for example, of certain types of random variation associated with sampling or sample under coverage or to adjust for probability of selection of household or of participants taking part to the study [72]. Ignoring clustering adjustments in analysis could lead to incorrect estimates of the standard error (SE) and type I error [73]. Although clustering has become common in most individual randomised trials, it is not always accounted for when assessing the intervention impact. In a review of 38 individually randomised clinical trials, the authors reported that only 11% adjusted for clustering and that, of the four that adjusted, three did not take into consideration all sources of clustering [74]. The following could lead to incorrect conclusions on the intervention impact [73]. Clustering adjustments in regression models can be introduced as random or fixed effects. In the Gambia and Tanzania larval control trials, the authors chose to include clustering adjustments as random effect during data analysis [9, 52]. In order to investigate the robustness of their modelling assumptions and because clusters were not allocated randomly, Maheu-Giroux & de Castro [9] performed additional analysis considering individual random effects, clusters fixed effects, and spatially-structured random effects. Further information on how to perform clustering adjustments during statistical analysis can be found in specialized publications [72].

Because data collected during a CRT could be over-dispersed or do not conform to normal distribution, it is advisable to consider using robust methods such as the negative binomial regression for data analysis [75, 76]. Also important is ensuring that selected statistical tests address the research question or the hypothesis and are compatible with the type and distribution of the data. Classifying variables as categorical, ordinal or continuous, to apply appropriate statistical tests could be helpful [69].

## Data collection

Data to collect during a larval control intervention has to be undertaken before and during the intervention and could include information from household, parasitological and entomological surveys. All these data put together, enable a good understanding of the performance of the trial and possible factors affecting the intervention.

### Baseline data collection and randomization

Collecting baseline data during an intervention is key because it enables to have well balanced groups before implementing the study. It is recommended to carry out baseline studies during a long period of about a year for example, to capture seasonal or temporal variations that could be responsible for misinterpretation of the intervention impact [4]. If malaria transmission is seasonal, baseline data collection can be undertaken during transmission seasons as was done in previous trials in the Gambia and Tanzania [9, 52]. Data collected describing the characteristics of each group must be presented in a detailed form, rather than presented as a text or in an incomplete form as reported for some CRT [77]. Using descriptive statistics for the presentation of baseline data rather than just displaying the significance of statistical tests between variables has the advantage that it allows detailed characterisation of the level of dispersion of each variable in each group and enables good comparison between groups [6, 52].

If at the end of baseline collections there is an imbalance between treated and untreated clusters’ characteristics meaning that randomization was unsuccessful, a selection of variables need to be undertaken to determine those that qualify for statistical adjustments. This situation usually occurs when a two-arm parallel random control trial design is used [64]. One way of solving the imbalance is to compare all baseline characteristics between the groups and to consider variables with statistically significant differences [78, 79]. This approach enables anticipation on statistical methods that can be used to control for differences or on ways of considering these differences when drawing conclusions about the study. Because this method ignores variables that are strongly correlated to the primary outcome but which are not significantly different between groups [78, 79], it is recommended in a first instance to consider variables displaying high correlation with the primary outcome as the most important [79]. A variable displaying a correlation coefficient greater or equal to 0.3 with the primary outcome is considered as suitable for statistical adjustment [79]. Methods used to control for imbalance between groups include study design adjustments (minimisation or stratification), matching of similar cluster in pairs or statistical adjustments [64, 69, 80]. Also if it is known in advance that a variable is strongly related to the prognosis, it might be important to consider adjusting for the variable during the study. For example, in a larval control intervention, it might be important to consider the usage rate of LLINs by the population or IRS applications as important covariates because they could influence the outcome of the study as observed in previous studies [6].

### Adult mosquito collections

In a malaria vector larval control intervention, mosquito sampling has to be undertaken both at the adult and the larval stages before and during the intervention to assess the efficacy of the intervention. Conducting adult mosquito surveys at baseline provides knowledge on whether mosquito populations are concentrated in certain geographical areas such as lowlands or if they are uniformly distributed. Conducting these surveys during intervention will determine whether the intervention is working or if there are hot spot areas where transmission persists. The major impact of a larval control intervention is the reduction of adult mosquito densities. The World Health Organization [4] recommends for adult mosquito collection, that a high number of places per cluster should be surveyed in order to capture all of the diversity in the environment and to measure the average community exposure. It is also important to carry repetitive collections to avoid large variation due to environmental factors such as rainfall [62]. According to Wilson et al. [62] using automated methods for mosquito collection such as light traps as done by Majambere et al. [52], reduces the risk of performance bias. On the other hand, the use of human landing catches or spray collection catches as reported in some trials [6, 8] could induce performance bias because the abundance of catches depends on the collector performance.

### Larval collections

Larval surveys have to include the prospection of all water pools, be it permanent or temporary breeding habitats. Also, standardized procedures have to be defined for larval collection. The use of a 350 ml dipper for estimating larval densities in breeding habitats is recommended. A standard number of dips should be undertaken per size of the water collection. According to the WHO [4] a dip could be enough for very small water collections such as a footprint or hoofprint; for larger water collection it is recommended to carry one dip per square meter for a maximum of 30 dips [4]. All these procedures have to be defined well in advance before conducting field collections. Also, for purpose of standardization, the personnel involved in larval survey have to be trained on how to distinguish between *Anopheles*, *Culex* and *Aedes* larvae, how to carry out larval collection, how to use field forms and on methods to physically or ecologically characterise breeding sites. After collection, the data should be introduced in a database for further analysis. If possible, a geographic information system (GIS) can be used to guide larval collection; this tool is now widely used in vector control trials [81–83]. The use of GIS technology has the advantage that it allows development of detailed maps and a clearly delineated system of numbering and logging of larval data and enables visualization of the data and integration with other data sources such as case management, adult mosquito collections or human behavioural surveys [4, 81, 83].

During data collection qualitative and quantitative data are collected from the field. It is recommended for qualitative variables not just to code them as present or absent but to register them according to their abundance. This will allow their transformation into categorical variables for different statistical comparisons. This method of registering qualitative field data was not always applied in most previous works [8, 52] whereas this could improve statistical data analysis and interpretation.

## Quality control analysis

### Quality control of the product

Following the World Health Organization recommendations, only WHOPES approved compounds should be used for larviciding efficacy or effectiveness trials [4] because they have satisfied a certain number of requirements established by WHOPES such as stability, potency, persistence in water bodies, reduced toxicity to human and non-target organisms. The full list of recommended larvicides is available in the WHOPES website (http://www.who.int/whopes/Mosquito_larvicides_28_July_2017.pdf). Before using a larvicide, it is recommended to carry laboratory tests to verify if the larvicide works well (whether the shipment or storage have not affected the larvicide efficacy) and also to confirm the range of concentrations to use for treatments. Quality control assessments need to be conducted before and during the intervention. Quality control assessments conducted during the intervention will provide information on the quality of the product during the process of storage, the susceptibility level of the target species, emergence of resistance and the influence of long term used of the compound on target and non-target organisms. Excluding few studies [84], the reporting of quality control assessment has not always been mentioned [5] whereas this information could be important to understand the limited impact of an intervention. Quality control tests have to be conducted with both field populations and laboratory colonies to detect more easily any variation. The methodology of conducting laboratory or semi-field tests could be found in WHO guidelines for laboratory and field testing of mosquito larvicides [85] and in some reference published works [84, 86, 87].

### Quality assurance of the implementation

Observing quality control guidelines increase the degree of confidence that the data collected is a true picture of what is really taking place on the field and is paramount for good evaluation of an intervention impact. Because of their stringency, these guidelines are not always followed in a high number of interventions. In a systematic review and critical appraisal of individualised random controlled trials conducted in China in 2004, the authors reported out of 307 studies analysed that 64.8% failed to report on methods of randomization, 82.4% did not mentioned blinding their participants or investigators, inadequate reporting of baseline data for a high proportion of studies and only 2.9% mentioned sample size calculations [77]. Similarly in a Cochrane review on larviciding studies conducted up to 2012, the authors reported high risk of bias for almost all studies conducted so far in Africa [5]. When conducting a larviciding intervention, it is important to ensure that methods used are greatly minimising the risk of bias and confounders. For this reason, defining a detailed monitoring strategy could be determinant. This includes:

(i). Having an independent group of assessors monitoring the treatment of sites different from those applying the larvicide on the field [88, 89];
(ii). Having within the personnel ensuring breeding site treatments, a person supervising activities and reporting on the successful completion of all activities (this group has to be blinded to sites chosen for random larval spot checks);
(iii). Conducting random larval spot-checks on a regular basis at least once every month 24 or 48 h after each treatment to verify if all breeding sites were targeted and treated. According to the World Health Organization [4], about 30 to 40 sites can be selected each month per cluster for random larval spot checks. It is also important to carry a follow-up in some sentinel sites;
(iv). If the larvicide has a long residual effect (˃ 3 weeks) such as Bti briquettes or insect growth regulators [4], regular inspections of sites once weekly to control the creation of new breeding habitats particularly during the rainy season need to be planned;
(v). Conducting regular adult mosquito collection in a high number of places to ensure that breeding sites treatment is effectively reducing adult mosquito biting in houses.

Quality assurance of the implementation can be associated to the use of a GIS system. This requires the acquisition of appropriate software, construction of a GIS database, acquisition of key material such as a server, maps, computers and mobile devices. The tracking of breeding habitats is undertaken using mobile devices. After each inspection, information collected from the field on breeding sites status (size, presence or absence of larvae, larval density, physical or ecological characteristics) are transferred in the GIS database. Reports of monitoring activities are generated regularly to assess the level of coverage of treatments. Untreated sites are also monitored similarly to assess seasonal fluctuations.

### Bias during larval control interventions

In their review of larviciding control interventions conducted so far across Africa, Tusting et al. [5] identified a certain number of biases that affected most larviciding studies conducted on the continent. Table 2 presents the most common biases that could affect a larval control trial and ways of avoiding or minimising these biases.

**Table 2:** A summary of common bias in larval control interventions and of ways for controlling these bias

No.	Bias	Corrective measures that could be applied
1	Random sequence generation (selection bias)	A central randomization procedure could be applied for random larval spot check. About 30 habitats randomly generated using a computer assisted programme out of the total number of habitats can be selected at least once monthly for each cluster by the programme manager including habitat ID and coordinates. This information is sent to the field supervisor for habitat inspection. Inspections have to be undertaken 1 or 2 days after larviciding treatments according to the timetable of treatments. For larvicides having a longer residual effect, inspections has also to be undertaken at 6-7 days intervals.
2	Allocation of concealment (selection bias)	Clusters have to be allocated as treated or untreated randomly. This random allocation can be done using a random table or a computer assisted programme.
3	Blinding of outcomes assessment (detection of bias)	Data collectors and the personnel processing the sample in the laboratory can be blinded to the intervention status.
4	Performance bias	Field applicators can be blinded for the sites choose for random larval spot check. Use automated methods for adult mosquito collection such as light traps. Use standardized measures for estimating larval densities.
5	Incomplete outcome data (attrition bias)	The sample size can be increased by adding 1 or 2 additional clusters per treatment group. This bias if not important can also be solved during statistical analysis.
6	Selective reporting (reporting bias)	All measured outcomes showing either a positive, non-significant or negative impact have to be reported as specified.
7	Baseline characteristics	Baseline data including entomological, ecological data and human behavioural data for each site has to be recorded before the intervention. Adjustment for a set of covariates can be applied to control for chance variations and improve precision of the impact estimates.
8	Contamination due to mosquito spillover	Consider a buffer zone of at least 1 km between treated and untreated clusters to minimise contamination due to mosquito spillover from untreated to treated zones. In addition, clusters have to be designed big enough so that the treatment is undertaken in the entire cluster but the evaluation is conducted only in the centre of the cluster (Fig. 1).
10	Incorrect data analysis	Use appropriate statistical methods and take into consideration during data analysis the clustering effect, covariates and confounding factors effects.
11	Sampling bias	Sampling has to be conducted in households selected randomly, use a large number of sites as possible for sampling, use automatic methods for sampling, carry mosquito collection during several days for each collection site to minimise bias due to rain or weather variations.

## Conclusions

Despite the progress registered during recent years, malaria vector control efforts across Africa are still affected by a high number of challenges including the spread of insecticide resistance, change in vector feeding and biting behaviours, outdoor malaria transmission and adaptation of mosquitoes to polluted environments. It is anticipated that additional control measures will be needed to improve control and elimination of the disease. The use of larval control in an integrated control approach could be crucial for managing insecticide resistance, for controlling outdoor biting mosquitoes or for malaria elimination particularly in urban settings where the intervention could be efficient and cost-effective. The present review summarises key parameters to take into consideration when planning and implementing larval control efficacy or effectiveness trials in order to improve the success of these interventions. The objective of the review was not to go into in-depth explanation of concepts and methods because this is available in several specialised documents, but to provide a document accessible to all desiring to undertake successful larval control interventions. Because poor study design, even in an area suitable for larviciding, will not achieve success, appropriate study design, thoroughness and good implementation are all required to drive interventions heading to malaria elimination across Africa.

## Abbreviations

Bti: *Bacillus thuringiensis israelensis*
CRT: Clusters randomised trials
GABA: Gamma-aminobutyric acid
IGR: Insect growth regulator
IRS: Indoor residual spraying
LLINs: Long-lasting insecticidal nets
MMF: Monomolecular surface films
WHO: World Health Organization

## References

[1] WHO. WHO global malaria programme, World Malaria Report. Geneva: WHO Press; 2015.

[2] WHO. World malaria report 2016. Geneva: World Health Organization; 2016. www.who.int/malaria. Accessed 13 dec 2016.

[3] WHO. WHO Global Malaria Programme World Malaria Report. Geneva: WHO Press; 2014.

[4] WHO. Larval source management a supplementary measure for malaria control. An operational manual. Geneva: World Health Organization; 2013.

[5] Tusting L, Thwing J, Sinclair D, Fillinger U, Gimnig J, Bonner K (2013). Mosquito larval source management for controlling malaria. Cochrane Database Syst Rev.

[6] Fillinger U, Ndenga B, Githeko A, Lindsay S (2009). Integrated malaria vector control with microbial larvicides and insecticide-treated nets in western Kenya: a controlled trial. Bull World Health Organ.

[7] Fillinger U, Lindsay S (2011). Larval source management for malaria control in Africa: myths and reality. Malar J.

[8] Geissbuhler Y, Kannady K, Chaki P, Emidi B, Govella N, Mayagaya V, et al. Microbial larvicide application by a large-scale, community-based program reduces malaria infection prevalence in urban Dar es Salaam, Tanzania. PLoS One. 2009;4(3):e5107. 10.1371/journal.pone.0005107 PMC2661378 19333402

[9] Maheu-Giroux M, Castro MC. Impact of community-based larviciding on the prevalence of malaria infection in Dar es Salaam, Tanzania. PLoS One. 2013;8(8):e71638. 10.1371/journal.pone.0071638 PMC3743749 23977099

[10] Afrane YA, Mweresa NG, Wanjala CL, Gilbreath TM, Zhou G, Lee M-C (2016). Evaluation of long-lasting microbial larvicide for malaria vector control in Kenya. Malar J.

[11] WHO. Report of the ninetheenth WHOPES working group meeting WHO/HQ Geneva 8–11 February 2016 Review of Veeralin LN, Vectomax GR, Bactivec SC. WHO control of neglected tropical diseases WHO pesticide evaluation scheme. Geneva: WHO Press; 2016.

[12] WHO. Interim position statement: the role of larviciding for malaria control in sub-Saharan Africa. World Health Organization Global Malaria Programme. Geneva: WHO Press; 2012.

[13] Tusting LS, Thwing J, Sinclair D, Fillinger U, Gimnig J, Bonner KE. Mosquito larval source management for controlling malaria. Cochrane Database Syst Rev. 2013:CD008923. 10.1002/14651858.CD008923.pub2 PMC4669681 23986463

[14] WHO. Monitoring of insecticide resistance in malaria vectors. World Health Organization Eastern Mediterranean Region (Cairo Egypt). Geneva: WHO Press; 2007.

[15] Yates W, Lindquist A, Mote D (1951). Suggestions for mosquito control in Oregon. Agricultural Experiment Station Bulletin.

[16] WHO. WHO position on DDT use in disease vector control under the Stockholm convention on persistent organic pollutants. WHO/HTM/RBM. Geneva: WHO Press; 2005.

[17] Van den Berg H (2009). Global status of DDT and its alternatives for use in vector control to prevent disease. Env Health Persp.

[18] Darwazeh HA, Mulla MS (1981). Pyrethrin tossits against mosquito larvae and their effects on mosquito fish and selected non target organisms. Mosq News.

[19] Mulla MS, Navvab-Gojrati H, Darwazeh HA (1978). Biological activity and longevity of new synthetic pyrethroids against mosquitoes and some nontarget insects. Mosq News..

[20] Bang YH, Sabuni IB, Tonn RJ. Integrated control of urban mosquitoes in Dar es Salaam using community sanitation supplemented by larviciding. East Afr Med J. 1975;52:578–88. 54247

[21] Hougard J, Agoua H, Yaméogo L, Akpoboua K, Sékétéli A, Dadzie K (1998). Blackfly control: what choices after onchocerciasis?. World Health Forum.

[22] Hougard J, Mbentengam R, Lochouarn L, Escaffre H, Darriet F, Barbazan P, Quillevere D (1993). Campaign against *Culex quinquefasciatus* using *Bacillus sphaericus*: results of a pilot project in a large urban area of equatorial Africa. Bull World Health Organ.

[23] Ghosh A, Chowdhury N, Chandra G (2012). Plant extracts as potential mosquito larvicides. Indian J Med Res.

[24] Maniafu BM, Wilber L, Ndiege IO, Wanjala CC, Akenga TA. Larvicidal activity of extracts from three *Plumbago* spp. against *Anopheles gambiae*. Mem Inst Oswaldo Cruz. 2009;104:813–7. 10.1590/s0074-02762009000600002 19876552

[25] Muema JM, Bargul JL, Njeru SN, Onyango JO, Imbahale SS. Prospects for malaria control through manipulation of mosquito larval habitats and olfactory-mediated behavioural responses using plant-derived compounds. Parasit Vectors. 2017;10:184. 10.1186/s13071-017-2122-8 PMC5392979 28412962

[26] Burton G (1967). Observations on the habits and control of *Culex pipiens fatigans* in Guyana. Bull World Health Organ.

[27] Darwazeh H, Fox R, Ramke D (1972). Efficacy of fortified petroleum oils as mosquito larvicides in irrigated pastures. Proc Calif Mosq Cont Assoc.

[28] Ramakrishnan S, Raghavan N, Krishnaswami A, Nair C, Basu P, Singh D (1960). National filaria control programme in India: a review (1955–59). Indian J Malariol.

[29] Ramakrishnan S, Sharma M, Kalra R (1960). Laboratory and field studies on the effectiveness of organophosphorus insecticides in the control of *C. fatigans*. Indian J Malariol.

[30] Djouaka R, Bakare A, Bankole H, Doannio J, Coulibaly O, Kossou H (2007). Does the spillage of petroleum products in *Anopheles* breeding sites have an impact on the pyrethroid resistance?. Malar J.

[31] Djouaka R, Bakare A, Bankole H, Doannio J, Kossou H, Akogbeto M (2007). Quantification of the efficiency of treatment of *Anopheles gambiae* breeding sites with petroleum products by local communities in areas of insecticide resistance in the Republic of Benin. Malar J.

[32] Nayar J, Ali A (2003). A review of monomolecular surface films as larvicides and pupicides of mosquitoes. J Vector Ecol.

[33] DRP M (1940). Problems concerning the efficiency of oils as mosquito larvicides. II. The spreading power of oils and methods of increasing it. Bull Entomol Res.

[34] Toms B (1950). Mosquito control: an investigation of natural surface films in relation to spreading of larvicidal oils upon water. Bull Entomol Res.

[35] Floore TG (2006). Mosquito larval control practices: Past and present. J Am Mosq Control Assoc.

[36] Mozley S, Butlerz M (1978). Effects of crude oil on aquatic insects of tundra ponds. Arctic.

[37] Lopes A, da Rosa-Osman S, Piedade M (2009). Effects of crude oil on survival, morphology, and anatomy of two aquatic macrophytes from the Amazon floodplains. Hydrobiologia.

[38] Mbare O, Lindsay S, Fillinger U (2014). Aquatain® mosquito formulation (AMF) for the control of immature *Anopheles gambiae sensu stricto* and *Anopheles arabiensis*: dose-responses, persistence and sub-lethal effects. Parasit Vectors.

[39] Reiter P (1978). The action of lecithin monolayers on mosquitoes II. Action on the respiratory structures. Ann Trop Med Parasitol.

[40] Levy R, Chizzonite J, Garrett W, Miller T (1981). Ground and aerial application of a monomolecular organic surface film to control salt-marsh mosquitoes in natural habitats of southwestern Florida. Mosq News..

[41] Kirst H (2010). The spinosyn family of insecticides: realizing the potential of natural products research. J Antibiotics.

[42] Darriet F, Duchon S, Hougard J (2005). Spinosad: a new larvicide against insecticide-resistant mosquito larvae. J Am Mosq Cont Ass.

[43] Romi RS, Proietti S, Di Luca M, Cristofaro M (2006). Laboratory evaluation of the bioinsecticide spinosad for mosquito control. J Am Mosq Control Assoc.

[44] Anderson JF, Ferrandino FJ, Dingman DW, Main AJ, Andreadis TG, Becnel JJ (2011). Control of mosquitoes in catch basins in Connecticut with *Bacillus thuringiensis israelensis*, *Bacillus sphaericus*, and spinosad. J Am Mosq Cont Ass..

[45] Hertlein MB, Mavrotas C, Jousseaume C, Lysandrou M, Thompson CD, Jany W (2010). A review of spinosad as a natural product for larval mosquito control. J Am Mosq Control Assoc.

[46] Davey R, George J, Snyder D (2001). Efficacy of a single whole-body spray treatment of spinosad against *Boophilus microplus* (Acari: Ixodidae) on cattle. Vet Parasitol.

[47] dos Santos Dias L, Macoris MLG, Andrighetti MTM, Otrera VCG, Dias AS, Bauzer LGSR (2017). Toxicity of spinosad to temephos-resistant *Aedes aegypti* populations in Brazil. PLoS One.

[48] Bielza P, Quinto V, Contreras J, Torné M, Martin A, Espinosa P (2007). Resistance to spinosad in the western flower thrips, *Frankliniella occidentalis* (Pergande), in greenhouses of south-eastern Spain. Pest Manag Sci.

[49] Zimmer CT, Garrood WT, Puinean AM, Eckel-Zimmer M, Williamson MS, Davies TGE (2016). A CRISPR/Cas9 mediated point mutation in the alpha 6 subunit of the nicotinic acetylcholine receptor confers resistance to spinosad in *Drosophila melanogaster*. Insect Biochem Mol Biol.

[50] Charles J, Nielsen-LeRoux C (2000). Mosquitocidal bacterial toxins: diversity, mode of action and resistance phenomena. Mem Inst Oswaldo Cruz.

[51] Orlova M, Smirnova T, Ganushkina L, Yacubovich V, Azizbekyan R (1998). Insecticial activity of *Bacillus laterosporus*. Appl Envir Microbiol.

[52] Majambere S, Pinder M, Fillinger U, Ameh D, Conway DJ, Green C (2010). Is mosquito larval source management appropriate for reducing malaria in areas of extensive flood in the Gambia? A cross-over intervention trial. Am J Trop Med Hyg.

[53] Pruszynski CA, Hribar LJ, Mickle R, Leal AL (2017). A large scale biorational approach using *Bacillus thuringiensis israeliensis* (strain AM65-52) for managing *Aedes aegypti* populations to prevent dengue, chikungunya and Zika transmission. PLoS One.

[54] Suman DS, Wang Y, Gaugler R. The insect growth regulator pyriproxyfen terminates egg diapause in the asian tiger mosquito, *Aedes albopictus*. PLoS One 2015;10(6):e0130499. 10.1371/journal.pone.0130499 PMC4474921 26090954

[55] Suman D, Wang Y, Bilgrami A, Gaugler R (2013). Ovicidal activity of three insect growth regulators against *Aedes* and *Culex* mosquitoes. Acta Trop.

[56] Kamal H, Khater E (2010). The biological effects of the insect growth regulators;pyriproxyfen and diflubenzuron on the mosquito *Aedes aegypti*. J Egypt Soc Parasitol.

[57] Lau K, Chen C, Lee H, Norma-Rashid Y, Sofian-Azirun M (2015). Evaluation of insect growth regulators against field collected *Aedes aegypti* and *Aedes albopictus* (Diptera: Culicidae) from Malaysia. J Med Entomol.

[58] Ohashi K, Nakada K, Ishiwatari T, Miyaguchi J, Shono Y, Lucas J (2012). Efficacy of pyriproxyfen-treated nets in sterilizing and shortening the longevity of *Anopheles gambiae* (Diptera: Culicidae). J Med Entomol.

[59] Ngufor C, N’Guessan R, Fagbohoun J, Odjo A, Malone D, Akogbeto M (2014). Olyset duo (a pyriproxyfen and permethrin mixture net): an experimental hut trial against pyrethroid resistant *Anopheles gambiae* and *Culex quinquefasciatus* in southern Benin. PLoS One.

[60] Harris C, Lwetoijera D, Dongus S, Matowo N, Lorenz L, Devine G (2013). Sterilising effects of pyriproxyfen on *Anopheles arabiensis* and its potential use in malaria control. Parasit Vectors.

[61] Flay B, Biglan A, Boruch R, Castro F, Gottfredson D, Kellam S (2005). Standards of evidence: criteria for efficacy, effectiveness and dissemination. Prev Sci.

[62] Wilson AL, Boelaert M, Kleinschmidt I, Pinder M, Scott TW, Tusting LS (2015). Evidence-based vector control? Improving the quality of vector control trials. Trends Parasitol.

[63] Zhou G, Afrane YA, Dixit A, Atieli HE, Lee M-C, Wanjala CL (2013). Modest additive effects of integrated vector control measures on malaria prevalence and transmission in western Kenya. Malar J.

[64] Imai K, King G, Nall C. The essential role of pair matching in cluster-randomized experiments, with application to the Mexican universal health insurance evaluation. Statist Sci. 2009;24:29–53.

[65] Gail M, Byar D, Pechacek T, Corle D. Aspects of statistical design for the community intervention trial for smoking cessation (COMMIT). Control Clin Trials 1992;13:16–25. 10.1016/0197-2456(92)90026-v 1315664

[66] Rutterford C, Copas A, Eldridge S (2015). Methods for sample size determination in cluster randomized trials. Int J Epidemiol.

[67] George L, Lenhart A, Toledo J, Lazaro A, Han WW, Velayudhan R (2015). Community-effectiveness of temephos for dengue vector control: a systematic literature review. PLoS Negl Trop Dis.

[68] Hayes R, Bennett S (1999). Simple sample size calculation for cluster-randomize trials. Int J Epidemiol.

[69] Logan PA, Gladman JRF, Avery A, Walker MF, Dyas J, Groom L (2004). Randomised controlled trial of an occupational therapy to increase outdoor mobility after stroke. BMJ.

[70] Hayes RJ, Moulton LH (2009). Cluster randomised trials.

[71] Killip S, Mahfoud Z, Pearce K. What is an intracluster correlation coefficient? Crucial concepts for primary care researchers. Ann Fam Med 2004;2:204–208. 10.1370/afm.141 PMC1466680 15209195

[72] Overview FGM. Of methods for analyzing cluster-correlated data. Boston: Harward School of Public Health; 2005.

[73] Kahan BC, Morris TP (2013). Assessing potential sources of clustering in individually randomised trials. BMC Med Res Methodol.

[74] Lee K, Thompson S (2005). Clustering by health professional in individually randomised trials. BMJ.

[75] Herbison P, Robertson MC, McKenzie JE. Do alternative methods for analysing count data produce similar estimates? Implications for meta-analyses. Syst Rev. 2015;4:163. 10.1186/s13643-015-0144-x PMC4650317 26577545

[76] Donaldson M, Sobolev B, Cook W, Janssen P, Khan K (2009). Analysis of recurrent events: a systematic review of randomised controlled trials of interventions to prevent falls. Age Ageing.

[77] Zhang D, Yin P, Freemantle N, Jordan R, Zhong N, Cheng KK (2008). An assessment of the quality of randomised controlled trials conducted in China. Trials.

[78] Fayers P, King M (2000). In reply to Berger "don't test for baseline imbalances unless they are known to be present?". Qual Life Res.

[79] Egbewale BE (2015). Statistical issues in randomised controlled trials: a narrative synthesis. Asian Pac J Trop Biomed.

[80] Balzer L, Petersen M, Van der Laan M (2012). Why match in individually and cluster randomized trials? University of California, Berkeley division of biostatistics working paper series.

[81] Dongus S, Nyika D, Kannady K, Mtasiwa D, Mshinda H, Fillinger U, et al. Participatory mapping of target areas to enable routine comprehensive larviciding of malaria vector mosquitoes in Dar es Salaam, Tanzania. Int J Health Geogr. 2007;6:37. 10.1186/1476-072X-6-37 PMC2025588 17784963

[82] Martin C, Curtis B, Fraser C, Sharp B (2002). The use of a GIS-based malaria information system for malaria research and control in South Africa. Health Place.

[83] Mlacha Y, Chaki P, Malishee A, Mwakalinga VM, Govella N, Limwagu A, et al. Fine scale mapping of malaria infection clusters by using routinely collected health facility data in urban Dar es Salaam, Tanzania. Geospat Health. 2017;12(494). 10.4081/gh.2017.494 28555474

[84] Majambere S, Lindsay S, Green C, Kandeh B, Fillinger U (2007). Microbial larvicides for malaria control in the Gambia. Malar J.

[85] WHO. Guidelines for laboratory and field testing of mosquito larvicides. World Health Organization communicable disease control, prevention and eradication WHO pesticide evaluation scheme. WHO/CDS/WHOPES/GCDPP/200513. Geneva: WHO Press; 2005.

[86] Dambach P, Louis V, Kaiser A, Ouedraogo S, Sié A, Sauerborn R, et al. Efficacy of *Bacillus thuringiensis* var. *israelensis* against malaria mosquitoes in northwestern Burkina Faso. Parasit Vectors. 2014;7:371. 10.1186/1756-3305-7-371 PMC4262221 25128297

[87] Nartey R, Owusu-Dabo E, Kruppa T, Baffour-Awuah S, Annan A, Oppong S, et al. Use of *Bacillus thuringiensis* var. *israelensis* as a viable option in an integrated malaria vector control programme in the Kumasi metropolis, Ghana. Parasit Vectors. 2013;6:116. 10.1186/1756-3305-6-116 PMC3637294 23607376

[88] Fillinger U, Kannady K, William G, Vanek M, Dongus S, Nyika D, et al. A tool box for operational mosquito larval control; preliminary results and early lessons from the urban malaria control Programme in Dar es Salaam. Malar J. 2008;7:20. 10.1186/1475-2875-7-20 PMC2259364 18218148

[89] Chaki PP, Govella NJ, Shoo B, Hemed A, Tanner M, Fillinger U, et al. Achieving high coverage of larval-stage mosquito surveillance: challenges for a community-based mosquito control programme in urban Dar es Salaam, Tanzania. Malar J. 2009;8(1):311. 10.1186/1475-2875-8-311 PMC2806382 20042071

